# Increased METTL3 expression and m^6^A RNA methylation may contribute to the development of dry eye in primary Sjögren’s syndrome

**DOI:** 10.1186/s12886-023-02988-0

**Published:** 2023-06-05

**Authors:** Jun Ma, Xiaotang Wang, Xue Yang, Xi Wang, Tongshan Tan, Hongping Fang, Yu Zhong, Qi Zhang

**Affiliations:** 1 The First Affiliated Hospital of Chongqing Medical University, Chongqing, China; 2 Chongqing Key Laboratory of Ophthalmology, Chongqing, China; 3 Chongqing Eye Institute, Chongqing, China; 4 Chongqing Branch of National Clinical Research Center for Ocular Diseases, Chongqing, China; 5 Department of Combination of Chinese and Western Medicine, The First Affiliated Hospital of Chongqing Medical University, Chongqing, China

**Keywords:** Primary Sjögren’s syndrome, Dry eye, N6-methyladenosine, METTL3

## Abstract

**Background:**

Primary Sjögren’s syndrome (pSS) is a chronic autoimmune disorder defined by xerostomia and keratoconjunctivitis sicca, and its etiology remains unknown. N6-methyladenosine (m^6^A) is the predominant posttranscriptional modification in eukaryotic mRNAs and is dynamically regulated by m^6^A regulators. Dysregulation of m^6^A modification is closely associated with several autoimmune disorders, but the role of m^6^A modification in pSS remains unknown. This study investigated the potential role of m^6^A and m^6^A-related regulators in pSS patients with dry eye.

**Methods:**

This cross-sectional study included forty-eight pSS patients with dry eye and forty healthy controls (HCs). Peripheral blood mononuclear cells (PBMCs) were isolated, and the level of m^6^A in total RNA was measured. The expression of m^6^A regulators was determined utilizing real-time PCR and western blotting. The serological indicators detected included autoantibodies, immunoglobulins (Igs), complement factors (Cs), and inflammatory indicators. Dry eye symptoms and signs were measured, including the ocular surface disease index, Schirmer’s test (ST), corneal fluorescein staining score (CFS), and tear break-up time. Spearman’s correlation coefficient was employed to assess the associations of m^6^A and m^6^A-related regulator expression with clinical characteristics.

**Results:**

The expression level of m^6^A was markedly increased in the PBMCs of pSS patients with dry eye compared to HCs (P _value_<0.001). The relative mRNA and protein expression levels of the m^6^A regulators methyltransferase-like 3 (METTL3) and YT521-B homology domains 1 were markedly elevated in pSS patients with dry eye (both P _value_<0.01). The m^6^A RNA level was found to be positively related to METTL3 expression in pSS patients (r = 0.793, P _value_<0.001). Both the m^6^A RNA level and METTL3 mRNA expression correlated with the anti-SSB antibody, IgG, ST, and CFS (all P _values_ < 0.05). The m^6^A RNA level was associated with C4 (r = -0.432, P _value_ = 0.002), while METTL3 mRNA expression was associated with C3 (r = -0.313, P _value_ = 0.030).

**Conclusions:**

Our work revealed that the upregulation of m^6^A and METTL3 was associated with the performance of serological indicators and dry eye signs in pSS patients with dry eye. METTL3 may contribute to the pathogenesis of dry eye related to pSS.

**Supplementary Information:**

The online version contains supplementary material available at 10.1186/s12886-023-02988-0.

## Introduction

Primary Sjögren’s syndrome (pSS) is a clinically common autoimmune disorder characterized by lymphocyte infiltration of exocrine glands (primarily the lacrimal and salivary glands) [1]. Glandular inflammation and tissue impairment eventually give rise to disturbances of secretory and clinical manifestations of dryness, including dry eye and xerostomia [2–4]. The pathogenesis of pSS is still poorly understood, and genetic and epigenetic factors have been considered to affect pSS development [5]. Existing research found irregular DNA methylation in pSS patients, and a genome-wide DNA methylation study recognized 1977 hypomethylated and 842 hypermethylated differentially methylated positions in pSS patients [6]. In addition to aberrant DNA methylation, increased miR-146a expression was also observed in peripheral blood mononuclear cells (PBMCs) from pSS patients [7]. A study showing histone hypoacetylation in pSS patients supports the concept that epigenetic factors contribute to the disease’s pathogenesis [8]. These studies show that epigenetic modifications promote the pathogenesis of pSS, but whether N6-methyladenosine (m^6^A) methylation is involved in epigenetic regulation in pSS with dry eye pathogenesis remains unknown.

m^6^A, the methylation modification at the sixth position of adenine bases in RNA, is the most common and evolutionarily conserved mRNA modification in eukaryotes [9]. m^6^A can affect RNA metabolism from some aspects, including mRNA splicing, mRNA stability and translation efficiency. The m^6^A modification is dynamically regulated by three groups of enzymes called methyltransferases (writer), demethylases (eraser) and binding proteins (reader) [10]. Methyltransferase-like 3 (METTL3), as the main RNA methyltransferase, forms a methyltransferase complex with its auxiliary partners methyltransferase-like 14 (METTL14) and Wilms tumor 1-associated protein (WTAP) to catalyze m^6^A modification [11]. It is well known that fat mass and obesity-associated protein (FTO) and alkylation repair homolog protein 5 (ALKBH5) are involved in removing m^6^A methylation [12]. In addition, m^6^A methylation is recognized via readers, including YTH and IGF2BP family proteins and affect the degradation and translation of downstream RNA [13, 14]. Recently, emerging studies have indicated that m^6^A modification is connected to some vital biological processes, especially inflammatory and autoimmune responses [15–17]. METTL3-mediated mRNA m^6^A methylation promotes dendritic cell activation [18]; B-cell-specific absence of METTL14 results in the B cell development defect [19]; WTAP promotes the differentiation of thymocytes [20]; FTO silencing inhibits macrophage polarization [21]. Moreover, ALKBH5 deficiency alleviates CD4 + T cell responses [22]; the deletion of YTHDF1 promotes the cross presentation of tumour antigens [23]. These results indicated that m^6^A may play a complicated role in pSS.

The objective of our research aimed to investigate the potential role of m^6^A modification in pSS with dry eye.

## Materials and methods

### Patients and controls

This cross-sectional study enrolled 48 pSS patients with dry eye from the First Affiliated Hospital of Chongqing Medical University from February 1, 2021, to April 1, 2022. These pSS patients accompanying with dry eye were diagnosed with the 2002 US-EU Consensus Group Criteria and the 2017 Dry Eye Workshop II Diagnostic Methodology Report strictly [24, 25]. The pSS patients were diagnosed by an ophthalmologist and a rheumatologist. The patients diagnosed as any other autoimmune diseases including rheumatoid arthritis (RA) and systemic lupus erythematosus, severe infection or taking immunomodulatory therapy previously were excluded. As the control group, forty healthy volunteers matched by gender and age were chosen. Approval for the present study was obtained from the Ethics Committee of the First Affiliated Hospital of Chongqing Medical University (2020 − 765).

### Measurement of serological indicators

Serum samples were routinely tested by the clinical pathology laboratory, including antinuclear antibodies (ANA), anti-SSA autoantibody, anti-SSB autoantibody, rheumatoid factor (RF), immunoglobulin A (IgA), immunoglobulin G (IgG), immunoglobulin M (IgM), complement 3 (C3), complement 4 (C4), C-reaction protein (CRP), and erythrocyte sedimentation rate (ESR).

### Ophthalmological evaluation

The dry eye symptoms and signs of pSS patients were examined via ocular surface disease index (OSDI) questionnaires. Patients with a score over 13 were diagnosed with symptomatic dry eye. Tear break-up time (TBUT), corneal fluorescein staining score (CFS), and Schirmer’s test (ST) were determined to examine the tear film. The TBUT and CFS evaluation was conducted in a room with low lighting. Using a fluorescein strip (Liaoning Meizilin Pharmaceutical Co. Ltd., Tianjin, China), fluorescein was applied to the lower conjunctival sac. The subjects were asked to blink, and the time before the first fault shown in the stained tear film was recorded. Under cobalt blue light, the CFS was measured utilizing the Oxford scale [26]. ST was carried out without topical anesthesia to assess the tear production of each individual. Filter paper (Liaoning Meizilin Pharmaceutical Co. Ltd., Tianjin, China) was applied for five mins. Readings were expressed as wetting millimeters.

### Extraction of lymphocytes

Peripheral blood samples (5 mL) were obtained, and then PBMCs were isolated by using lymphocyte separation medium (LDS1075, TBD, China) within two hours. The isolated PBMCs were divided into several small tubes and then separately preserved in TRIzol reagent (Roche, Switzerland) and RIPA buffer (P0013B, Beyotime, China). The isolated PBMCs samples were stored in liquid nitrogen until use.

### RNA isolation and real-time qPCR

Total RNA was isolated from the PBMCs of all participants with TRIzol reagent based on the manufacturer’s protocol. A spectrophotometer (NP80-Touch, Agilent Technologies) was employed to assess the purity and concentration of total RNA. Samples containing 1.0 µg of RNA were purified and synthesized into cDNA using RT Master Mix for qPCR (HY-K0511, MCE, USA). The cDNA was amplified employing SYBR Green qPCR Master Mix (HY-K0522, MCE, USA), and the fluorescent signal was monitored by an Applied Biosystems 7500 System. All primer sequences utilized in this work are displayed in Table 1. The relative expression of m^6^A methylation-related genes was normalized to the internal reference and assessed through the 2^−ΔΔCT^ method.

**Table 1:** Primers used in this study

Gene	Forward Primer	Reverse Primer
GAPDH	GGA GCG AGA TCC CTC CAA AAT	GGC TGT TGT CAT ACT TCT CAT GG
METTL3	TTG TCT CCA ACC TTC CGT AGT	CCA GAT CAG AGA GGT GGT GTA G
METTL14	AGT GCC GAC AGC ATT GGT G	GGA GCA GAG GTA TCA TAG GAA GC
WTAP	ACC TCT TCC CAA GAA GGT TCG	GAT CTG TGT ACT TGC CCT CCA
ALKBH5	CGC TGC CGC CGA ACC TTA C	GGA TGC CGC TCT TCA CCT TGC
FTO	CCA GGG TTG GGA TGG GTT CA	CGC TGA CCT GTC CAC CAG AT
YTHDF1	AGC ACA GAG CAC GGC AAC AAG	CCA TTG ACG CTG AAG AGC AGG TAG
YTHDF2	CAG ACA CAG CCA TTG CCT CCA C	AGA ACC AGC CTG AGA CTG TCC TAC

### Western blot analysis

The preserved protein samples from the PBMCs were isolated with RIPA lysis buffer (P0013B, Beyotime, China) containing a protease inhibitor cocktail (ST507, Beyotime, China), and the content was quantified by the BCA assay (P0012S, Beyotime). Lysates with 10 µg of total protein were isolated on 4–20% SDS‒PAGE gels and subsequently placed onto polyvinylidene fluoride membranes, according to the conventional method. After blocking with fat-free milk for one hour, the membranes were incubated with primary antibody overnight at room temperature. Primary antibodies against GAPDH (ab181602, Abcam, USA), METTL3 (ab195352, Abcam, USA), and YTHDF1 (ab220162, Abcam, USA) were used. Then, the membranes were rinsed with washing buffer three times and incubated for one hour with the secondary antibody (ab97051, Abcam, USA). The protein blots were visualized with an enhanced chemiluminescence kit (P0018FS, Beyotime, China). Protein expression was semi quantitatively analyzed with ImageJ software.

### Quantification of RNA m^6^A

The m^6^A RNA methylation level was determined by utilizing the EpiQuik m^6^A RNA Methylation Quantification Kit (Colorimetric, Epigentek, USA) following the manufacturer’s protocols. The relative abundance of m^6^A was measured and calculated by the absorbance detected by a microplate spectrophotometer (Varioskan Lux, Thermo) at 450 nm.

### Statistical analysis

GraphPad Prism 8.0 (GraphPad Software) was used for all statistical analyses. Numerical data with a normal distribution are indicated as the mean ± standard deviation (SD), and differences between the two groups were studied by a two-tailed Student’s t test. Numerical data with skewed distributions are indicated as the median (25th percentile-75th percentile), and differences between the two groups were examined by the Mann–Whitney U test. Categorical data are indicated as percentages and frequencies. The correlation was evaluated by Spearman’s correlation coefficient. A P _value_ < 0.05 was regarded as statistically significant.

## Results

### Characteristics of pSS patients and controls

This study included 48 pSS patients and 40 HCs matched for age and gender. The mean age of 48 pSS patients was 45.67 ± 12.43 during our study. Demographic, laboratory, and clinical characteristics of pSS patients and HCs are shown in Table 2. Anti-SSA antibodies were positive in 35 of 48 (72.92%) patients, and anti-SSB antibodies were positive in 21 of 48 (43.75%) patients. There were significant differences in RF, IgA, IgG, C3, C4, and ESR levels between pSS patients and HCs (P _value_<0.001; P _value_<0.001; P _value_<0.001; P _value_<0.001; P _value_=0.025; P _value_<0.001; respectively), but no significant difference was found in IgM and CRP levels between them (P _value_=0.919; P _value_=0.187; respectively).

**Table 2:** Clinical characteristics data

	pSS patients	Health controls	P value	
Number, n	48	40	-	
Age, years	45.67 ± 12.43	46.28 ± 8.61	0.794	
Sex, female (%)	44 (91.67)	36 (90.00)	0.787	
Serological indicators				
ANA, n (%)	43 (89.58)	0(0.00)	<0.001	
Anti-SSA+, n (%)	35 (72.92)	0(0.00)	<0.001	
Anti-SSB+, n (%)	21 (43.75)	0(0.00)	<0.001	
RF, IU/mL	25.00 (20.00–82.38)	11.00 (7.00–18.50)	<0.001	
IgA, g/L	2.65 (2.14–3.49)	1.83 (1.41–2.46)	<0.001	
IgG, g/L	16.85 (13.13–21.00)	11.70 (9.48–12.68)	<0.001	
IgM, g/L	1.26 (0.99–2.00)	1.39 (1.01–1.97)	0.919	
C3, g/L	0.78 (0.69–0.87)	0.90 (0.83–0.95)	<0.001	
C4, g/L	0.18 (0.14–0.21)	0.21 (0.17–0.23)	0.025	
CRP, mg/L	1.83 (1.17–3.23)	1.55 (1.27–2.63)	0.187	
ESR, mm/h	29.50 (15.00–61.75)	9.00 (6.00–15.50)	<0.001	
Dry eye signs and symptoms				
OSDI	25.00 (16.00–41.50)	8.00 (6.00–10.75)	<0.001	
ST, mm	2.00 (1.00–3.75)	17.00 (13.50–22.00)	<0.001	
TBUT, s	2.00 (1.00–2.00)	11.00 (9.00–13.00)	<0.001	
CFS	4.50 (3.00–6.75)	0.00 (0.00–0.00)	<0.001	
Clinical manifestations, n (%)				
Dry mouth	41 (85.42)	0 (0)	-	
Fever	20 (41.67)	0 (0)	-	
Fatigue	15 (31.25)	0 (0)	-	
Arthralgias	22 (45.83)	0 (0)	-	
Respiratory system involvement	18 (37.50)	0 (0)	-	
Renal involvement	17 (35.42)	0 (0)	-	

Data are presented as mean ± standard deviation (SD) median (25% percentile-75% percentile) or for continuous data and number (percentage) for categorical variable. pSS: Primary Sjögren’s syndrome; ANA, antinuclear antibodies; Anti-SSA, anti-SSA antinuclear antibodies; Anti-SSB, anti-SSB antinuclear antibodies; RF, rheumatoid factor; Igs: immunoglobulins; Cs: complement factors; CRP, C-reactive protein; ESR, erythrocyte sedimentation rate; OSDI: ocular surface disease index, ST: the Schirmer’s test; CFS: corneal fluorescein staining score; TBUT: tear break-up time

### Increased m^6^A levels and aberrant m^6^A regulators in pSS patients with dry eye

**Fig. 1:**
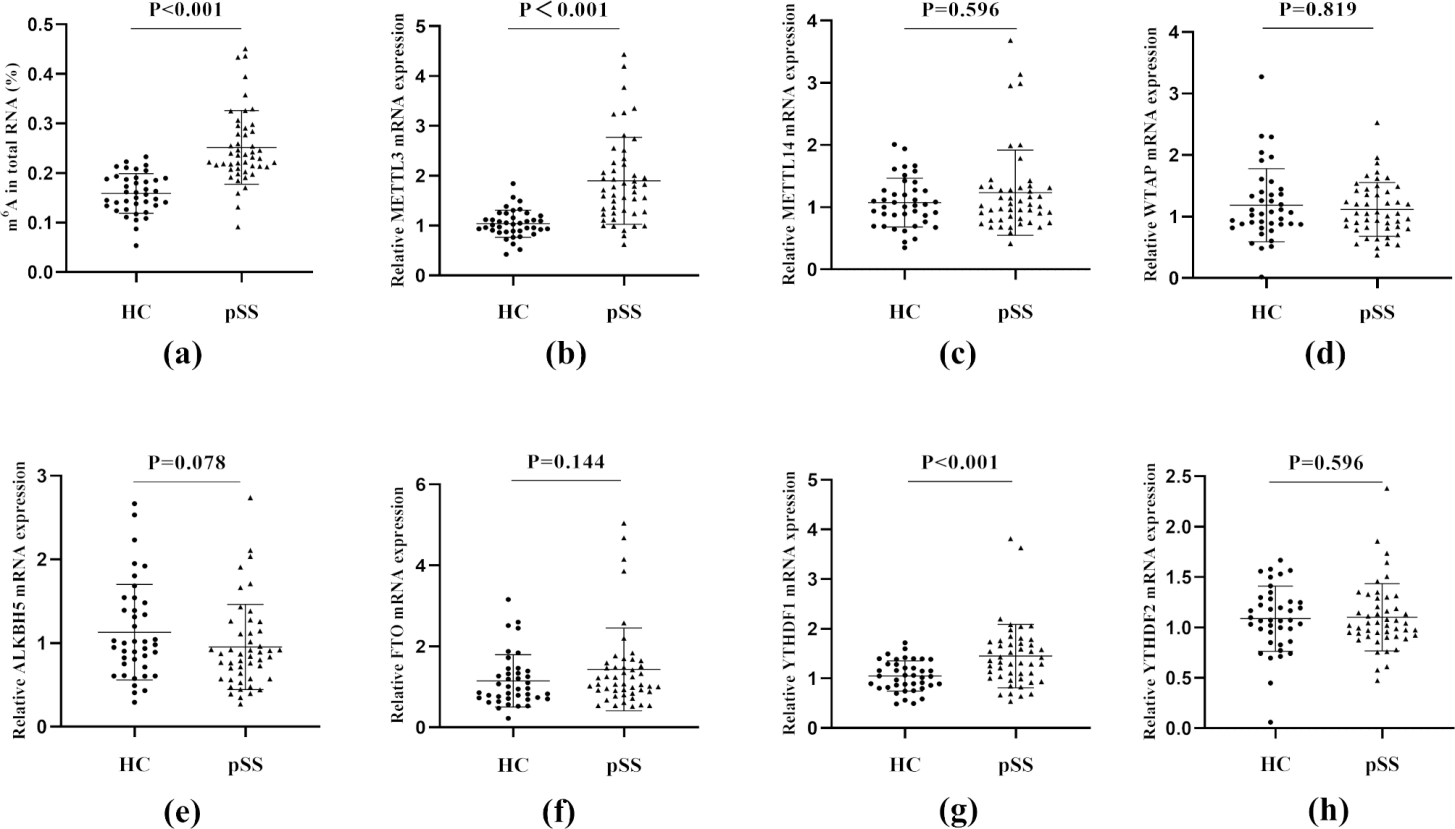
The increased m^6^ A RNA levels and elevated METTL3 and YTHDF1 mRNA levels in the PBMCs of pSS patients with dry eye. **(a-h)** The RNA levels of m^6^A/ METTL3/ METTL14/ WTAP/ ALKBH5/FTO/ YTHDF1/ YTHDF2 in PBMCs from pSS patients with dry eye and HCs. (Data are means ± SD for 48/pSS and 40/HCs; P value by Mann–Whitney U test (a-f, h) and unpaired Student’s t-test **(g)**)

**Fig. 2:**
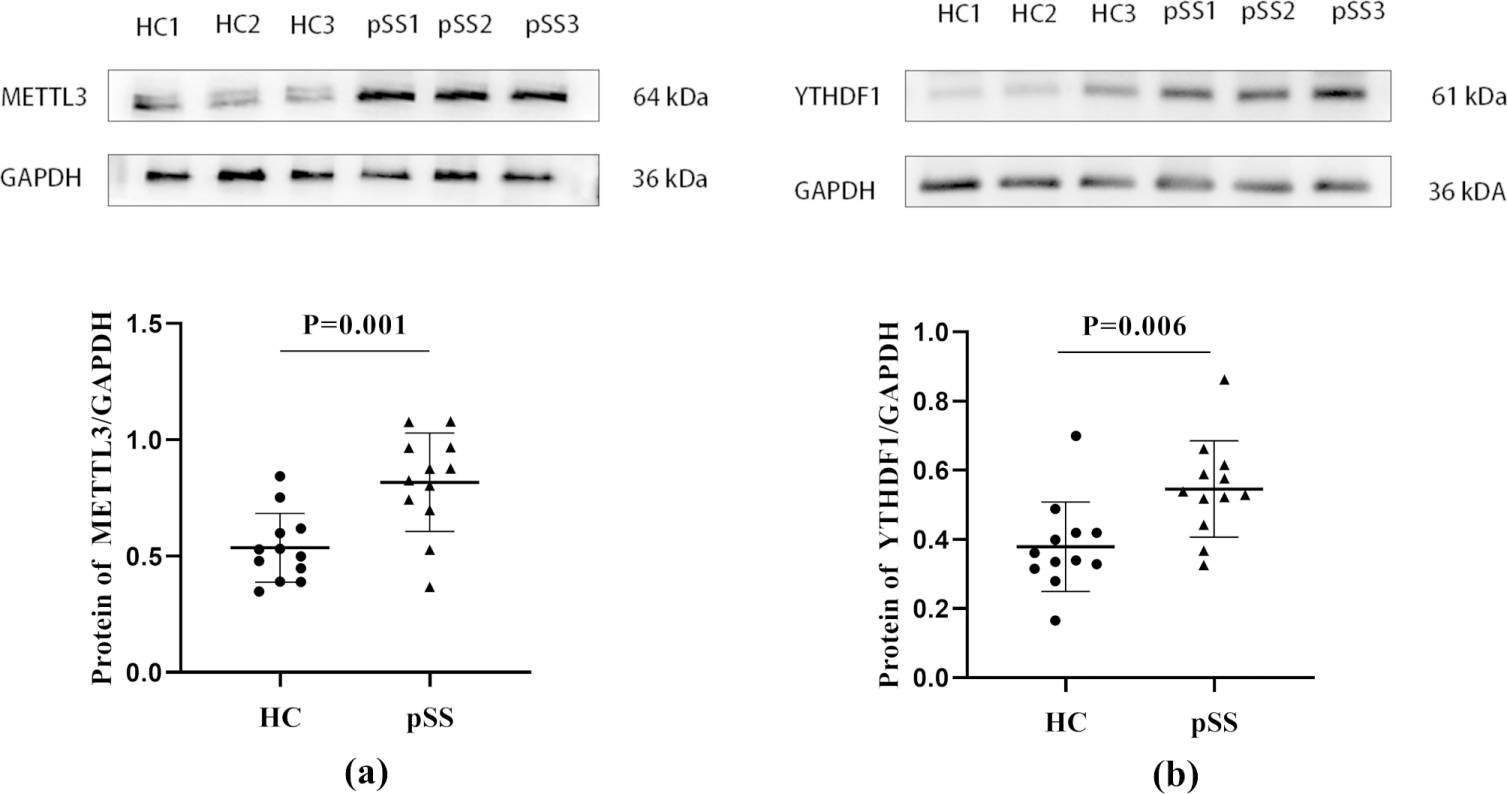
The elevated METTL3 and YTHDF1 protein levels in the PBMCs of the pSS patients with dry eye. **(a)** The protein level of METTL3 in PBMCs from pSS patients with dry eye and HCs. Upper panel, representative western blotting images of METTL3. Lower panel, quantification of the relative expression of METTL3. **(b)** The protein level of YTHDF1 in PBMCs from pSS patients with dry eye and HCs. Upper panel, representative western blotting images of YTHDF1. Lower panel, quantification of the relative expression of YTHDF1. (Data are means ± SD for 12 samples; P value by unpaired Student’s t-test (a-b))

To preliminarily investigate the role of m^6^A levels in pSS with dry eye, we first analyzed the m^6^A RNA level in the PBMCs of patients with this condition. The m^6^A expression level in pSS patients was increased compared with that in HCs (P _value_<0.001) (Fig. 1a). We then determined the mRNA and protein expression levels of several enzymes participating in m^6^A modification. The mRNA levels of METTL3 and YTHDF1 were markedly increased in pSS patients compared with HCs, while there was no significant difference in METTL14, WTAP, ALKBH5, FTO, or YTHDF2 between the two groups (P _value_=0.596; P _value_=0.819; P _value_=0.078; P _value_=0.144; P _value_=0.596; respectively) (Fig. 1b-h). The protein expression levels of METTL3 and YTHDF1 in pSS patients were increased compared to those in HCs (P _value_=0.001, P _value_=0.006, respectively) (Fig. 2a-b), which is consistent with the mRNA expression results.

### The correlation of m^6^A levels with METTL3 mRNA expression in pSS patients with dry eye

**Fig. 3:**
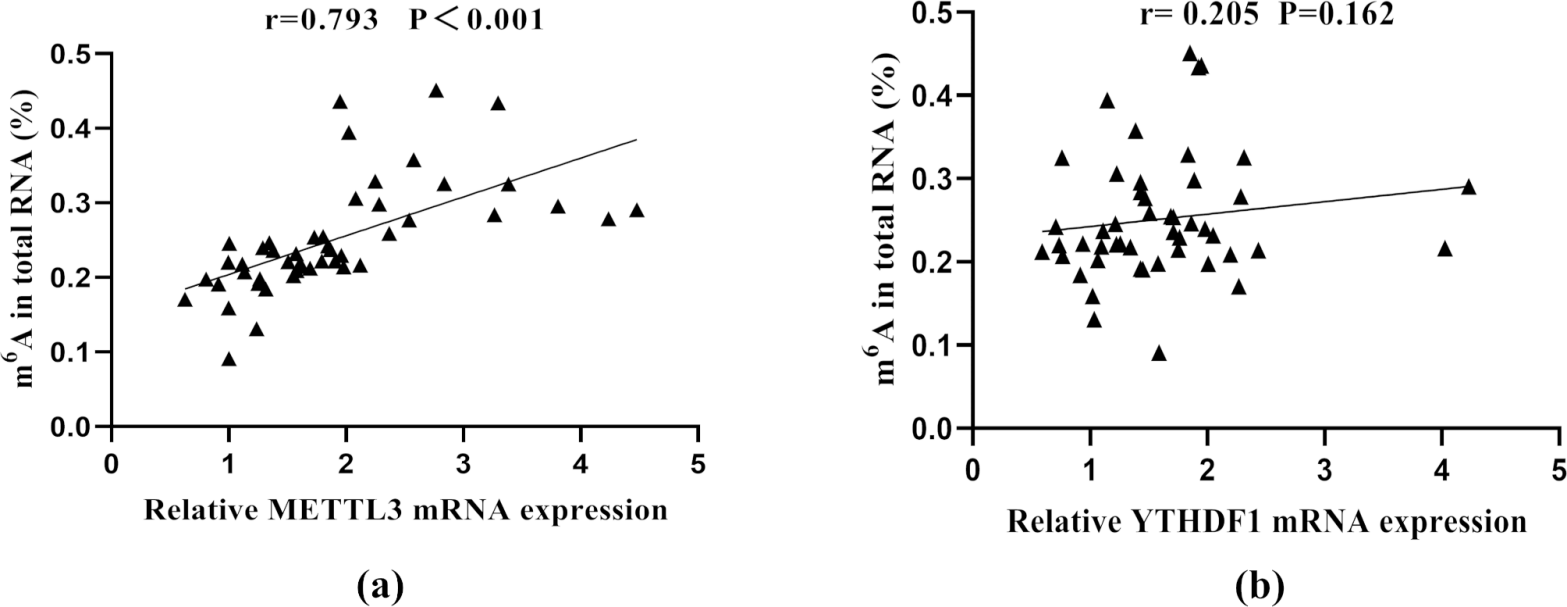
The positive correlation of m^6^ A RNA levels with the METTL3 mRNA level in the PBMCs of pSS patients with dry eye. **(a)** The correlation analyses between m^6^A and METTL3 mRNA levels in PBMCs from pSS patients with dry eye. **(b)** The correlation analysis among m^6^A and YTHDF1 mRNA levels in PBMCs from pSS patients with dry eye

We hypothesized that the aberrant m^6^A expression level in pSS patients with dry eye was caused by the dysregulation of m^6^A regulators. Thus, we analyzed the correlation between the m^6^A expression level and related m^6^A regulator expression. The m^6^A expression level in pSS patients with dry eye was positively associated with increased METTL3 expression (r = 0.793, P _value_<0.001) (Fig. 3a). There was no association between m^6^A and YTHDF1 (r = 0.205, P _value_=0.162) (Fig. 3b). Moreover, no association was found for m^6^A and METTL3 in HCs (r = -0.116, P _value_=0.467) (Supplemental Fig. 1a).

### Clinical significance of increased m^6^A levels and METTL3 expression in pSS patients with dry eye

**Fig. 4:**
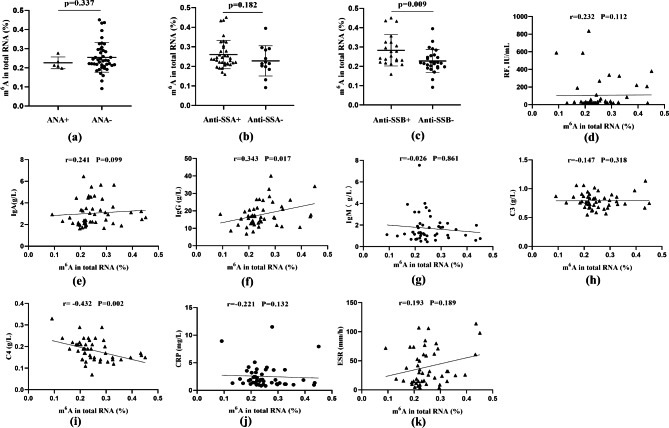
The correlation analyses of m^6^ A RNA levels in PBMCs with the serological indicators of pSS patients with dry eye. **(a-c)** The RNA level of m^6^A in PBMCs from ANA/anti-SSA/anti-SSB-positive patients with dry eye and respective-negative patients. **(d-k)** The correlation analysis between m^6^A and RF/IgA/IgG/IgM/C3/C4/CRP/ESR levels in pSS patients with dry eye, respectively. (Data are means ± SD for 48 samples; P value by Mann–Whitney U test (a-b) and unpaired Student’s t-test (c))

To identify the clinical significance of increased m^6^A levels in pSS patients with dry eye, we analyzed the relationship between the m^6^A level and the performance of serological indicators in these patients, including ANA, anti-SSA autoantibody, anti-SSB autoantibody, RF, IgA, IgG, IgM, C3, C4, CRP, and ESR expression levels. We found that the m^6^A level was remarkably higher in anti-SSB-positive patients than in anti-SSB-negative patients (P _value_=0.009) but was not statistically correlated with ANA and anti-SSA autoantibodies (P _value_=0.337; P _value_=0.182; respectively) (Fig. 4a-c). In addition, the m^6^A level was positively associated with the IgG expression level (r = 0.343, P _value_= 0.017) and negatively associated with the C4 expression level (r = -0.432, P _value_ = 0.002), but there was no correlation with the levels of RF, IgA, IgM, C3, CRP, and ESR (r = 0.232, P _value_ = 0.112; r = 0.241, P _value_ = 0.099; r = -0.026, P _value_ = 0.861; r = -0.147, P _value_ = 0.318; r = -0.221, P _value_ = 0.132; r = 0.193, P _value_ = 0.189; respectively) (Fig. 4d-k).

To further study the potential role of aberrant m^6^A levels in ocular surface damage, we analyzed the relationships between m^6^A levels and dry eye symptoms and signs in pSS patients. As shown in Table 3, a positive correlation between the m^6^A level and CFS was observed (r = 0.389, P _value_ = 0.006). The m^6^A level was found to be negatively associated with the ST value (r = -0.364, P _value_ = 0.011), whereas no association was found for OSDI and FBUT (r = -0.008, P _value_ = 0.959; r = -0.203, P _value_ = 0.167; respectively).

**Table 3:** Correlation analyses of m6A and METTL3 levels with dry eye signs and symptoms in pSS patients

Dry eye signs and symptoms	METTL3		m^6^A	
	Spearman r	P	Spearman r	P
OSDI	-0.066	0.658	-0.008	0.959
ST	**-0.358**	**0.013**	**-0.364**	**0.011**
TBUT	0.249	0.087	-0.203	0.167
CFS	**0.456**	**0.001**	**0.389**	**0.006**

METTL3: methyltransferase-like 3; m^6^A: N6-methyladenosine; OSDI: ocular surface disease index, ST: the Schirmer’s test; CFS: corneal fluorescein staining score; TBUT: tear break-up time

**Fig. 5:**
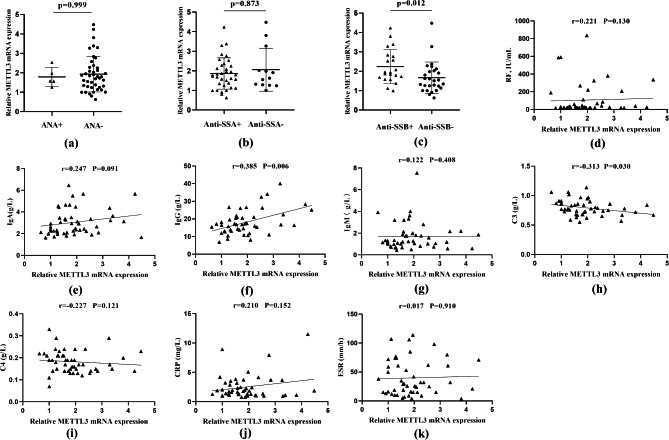
The correlation analyses of METTL3 mRNA expression in PBMCs with the serological indicators of pSS patients with dry eye. **(a-c)** The RNA level of METTL3 in PBMCs from ANA/anti-SSA/anti-SSB-positive patients with dry eye and respective-negative patients. **(d-k)** The correlation analysis between METTL3 and RF/IgA/IgG/IgM/C3/C4/CRP/ESR levels in pSS patients with dry eye, respectively. (Data are means ± SD for 48 samples; P value by Mann–Whitney U test (a-c))

We determined the relationships between the expression levels of METTL3 and clinical characteristics. As shown in Fig. 5; Table 3, METTL3 expression was significantly enhanced in anti-SSB-positive patients (P _value_= 0.012). A positive correlation of METTL3 expression with IgG (r = 0.385, P _value_ = 0.006) and CFS (r = 0.456, P _value_= 0.001) and a negative correlation of METTL3 expression with C3 (r = -0.313, P _value_= 0.030) and ST values (r = -0.358, P _value_= 0.013) were found. There was no correlation between METTL3 expression and RF, IgA, IgM, C4, CRP, ESR, OSDI, or FBUT in pSS patients. (r = 0.221, P _value_ = 0.130; r = 0.247, P _value_ = 0.091; r = 0.122, P _value_ = 0.408; r = -0.227, P _value_ = 0.121; r = 0.210, P _value_ = 0.152; r = 0.017, P _value_ = 0.910; r = -0.066, P _value_ = 0.658; r = 0.249, P _value_ = 0.087, respectively).

## Discussion

pSS is a chronic inflammatory autoimmune disease and is characterized by exocrine gland impairment, such as in the salivary and lacrimal glands, which could result in dry mouth and eye [1]. m^6^A, as the most abundant modification in mRNA, is receiving increasing attention and has been found to function in viral infections and some autoimmune diseases in recent years [27, 28]. The potential role of m^6^A modification in pSS patients with dry eye remains largely unknown. Therefore, our work aimed to examine the levels of m^6^A modification and m^6^A-related regulator expression in the lymphocytes of pSS patients with dry eye and analyze their correlation with clinical characteristics. Our findings revealed that the expression level of the m^6^A modification and the mRNA and protein expression of METTL3 and YTHDF1 were all increased in pSS patients with dry eye. Moreover, the m^6^A level was positively correlated with METTL3 in pSS patients with dry eye. The correlation analyses indicated that m^6^A and METTL3 expression were correlated with anti-SSB antibody, IgG, complement, CFS, and ST. These results suggest a complicated role of m^6^A modifications in pSS with dry eye.

An increasing number of experiments have stated that the dysregulation of global m^6^A abundance and the aberrant expression of m^6^A regulators might be associated with various autoimmune disorders. Wang et al. [29] found that METTL3 was elevated in PBMCs from RA patients. In vitro experiments showed that METTL3 upregulation in macrophages increased the overall m^6^A content and that METTL3-related m^6^A modification was correlated with the secretion of inflammatory factors. Song et al. [30] revealed that METTL3 mutations might be a pivotal susceptibility factor for autoimmune thyroid disease. These findings indicated that aberrant m^6^A modification is a new regulatory mechanism in autoimmune disease. Recently, Cheng and her colleagues reported the downregulated expression of RNA-binding motif protein X-linked, ALKBH5, YTH domain-containing protein 2, and YTHDF1 in the peripheral blood samples of pSS patients [31]. The reasons for this conflicting finding might be the distinctions in the disease severity of selected patients and the investigated cell type.

The US-EU Consensus Group has suggested that one of the criteria for the diagnosis of pSS is the occurrence of anti-SSB or anti-SSA autoantibodies [24]. Previous results indicated that the anti-SSB antibody displays relatively better specificity for diagnosis [1]. The association of the expression of m^6^A and METTL3 with anti-SSB antibodies (not anti‐SSA antibodies) might be due to the better specificity of anti-SSB antibodies. These findings further revealed that m^6^A modification might contribute to the pathogenesis of pSS with dry eye. Excessive immunological and inflammatory responses are crucial features of pSS, which can be evaluated by the expression levels of IgG and complement C3 and C4 [32, 33]. Wang et al. [29] revealed that increased levels of METTL3 correlated with CRP and ESR in rheumatoid arthritis, which is similar to our findings. Our results indicated that the elevated expression of m^6^A and METTL3 correlated with serological immune indicators in pSS patients with dry eye. Huang et al. [19] reported that m^6^A-deficient mice exhibited the impairment of B cells activation and autoantibodies secretion, we speculate that aberrant m^6^A modification may lead to the excessive secretion of anti-SSB antibodies and IgG in B cells and the release of complements in pSS. m^6^A and METTL3 levels might be used as potential laboratory parameters to evaluate the systemic immune condition of pSS patients in clinical practice. Moreover, m^6^A methylation could be a potential candidate for the epigenetic-based treatment of pSS.

Previous studies have focused on the correlation between ocular manifestations of autoimmune diseases and aberrant m^6^A modification. Zhu et al. [34] reported that m^6^A expression was significantly increased in extraocular muscle samples from Graves’ ophthalmopathy patients, which suggests that dysregulated m^6^A regulators may lead to the upregulated expression of genes related to the immune response and inflammatory process, thereby leading to ocular autoimmune diseases. We observed a significant correlation of m^6^A and METTL3 expression with certain signs of dry eye when assessing tear secretion and ocular surface damage, which indicates that aberrant m^6^A modification may contribute to the pathogenesis of dry eye in pSS.

However, there are a few limitations of our work. First, our work included only patients from the Department of Ophthalmology, which might lead to selection bias. Further studies could enroll pSS patients from other clinical departments and compare the correlation of m^6^A expression with other clinical features of pSS. Second, although increased METTL3 and m^6^A levels influenced the immune response in pSS, the precise regulatory mechanism is unknown. Our results suggest that aberrant m^6^A modification and METTL3 expression are likely to contribute to pSS pathogenesis, but further in vivo experimental studies are needed. Third, although PBMCs can characterize some disease, the data will be stronger if performed in conjunctiva impression cytology to reflect ocular changes of pSS patients.

## Conclusions

In summary, we found an increased level of m^6^A methylation and elevated expression of MEETL3 in PBMCs from pSS patients with dry eye. Our work revealed that upregulation of m^6^A and METTL3 was related to the manifestation of serological indicators and dry eye signs in pSS patients with dry eye, which indicates that METTL3 may contribute to the pathogenesis of dry eye related to pSS.

## Electronic supplementary material

Below is the link to the electronic supplementary material.

Additional file 1: Fig. 1. The correlation analyses of m^6^A RNA level with the METTL3 mRNA level in the PBMCs of health controls. Fig. 2. Representative raw images showing METTL3 and YTHDF1 expression in the different group

## Data Availability

The data used to support the findings of this study are available upon request to the corresponding author.

## Abbreviations

pSS: Primary Sjögren’s syndrome
m^6^A: N6-methyladenosine
HCs: Healthy controls
PBMCs: Peripheral blood mononuclear cells
Igs: Immunoglobulins
Cs: Complement factors
OSDI: Ocular surface disease index, ST:the Schirmer’s test
CFS: Corneal fluorescein staining score
TBUT: Tear break-up time
METTL3: Methyltransferase-like 3
YTHDF1: YT521-B homology domains 1
METTL14: Methyltransferase-like 14
WTAP: Wilms tumor 1-associated protein
FTO: Obesity-associated protein
ALKBH5: Alkylation repair homolog protein 5
YTHDF2: YT521-B homology domains 2
RA: Rheumatoid arthritis.
