# Supplementary material for: Increased METTL3 expression and m6A RNA methylation may contribute to the development of dry eye in primary Sjögren’s syndrome
Source: BMC Ophthalmol. 2023 Jun 5;23:252. doi: 10.1186/s12886-023-02988-0 (PMC10243060; doi:10.1186/s12886-023-02988-0)

**(a)**

**Supplemental Figure 1.**

**The correlation analyses of m^6^A RNA level with the METTL3 mRNA level in the PBMCs of health controls**


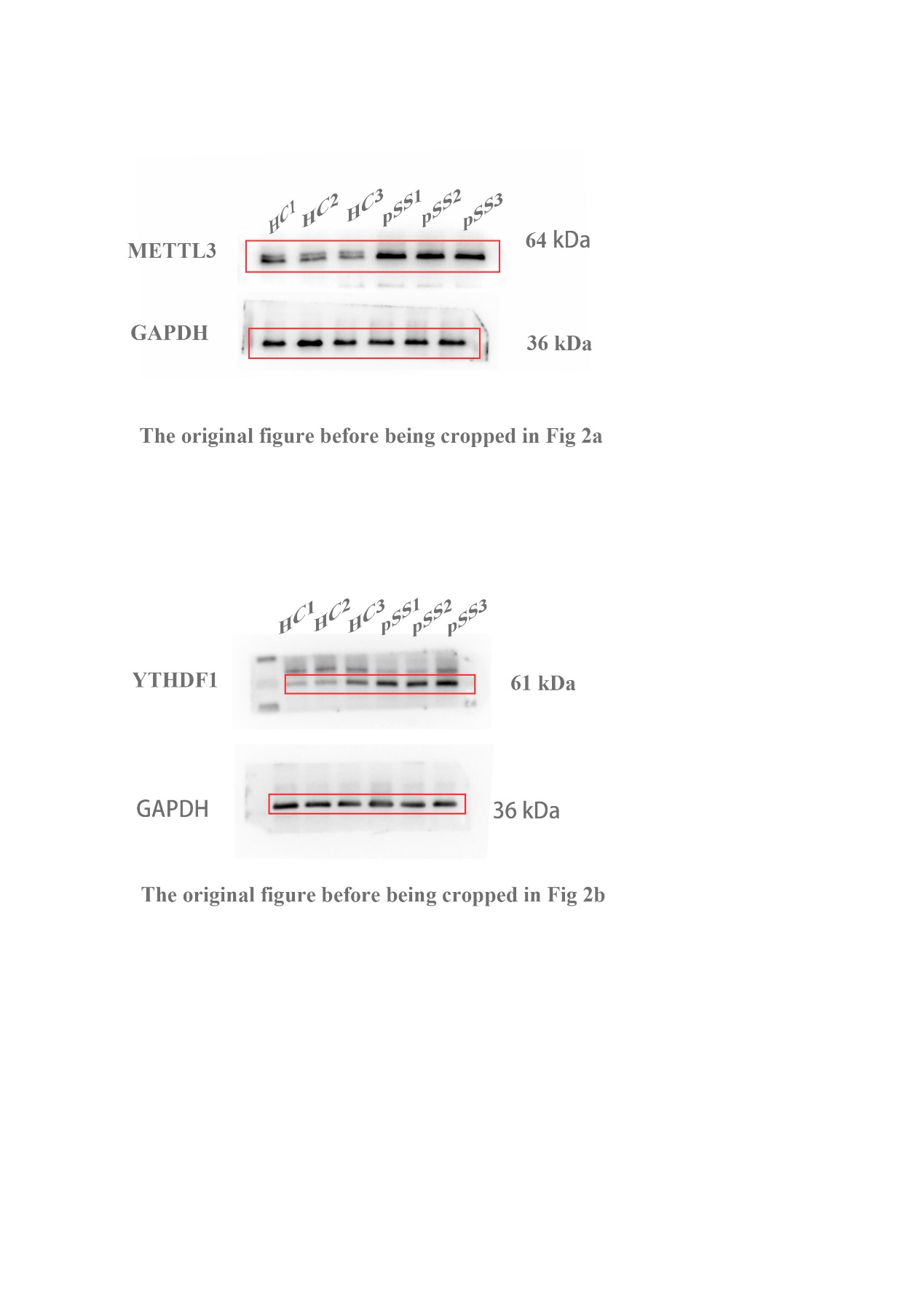


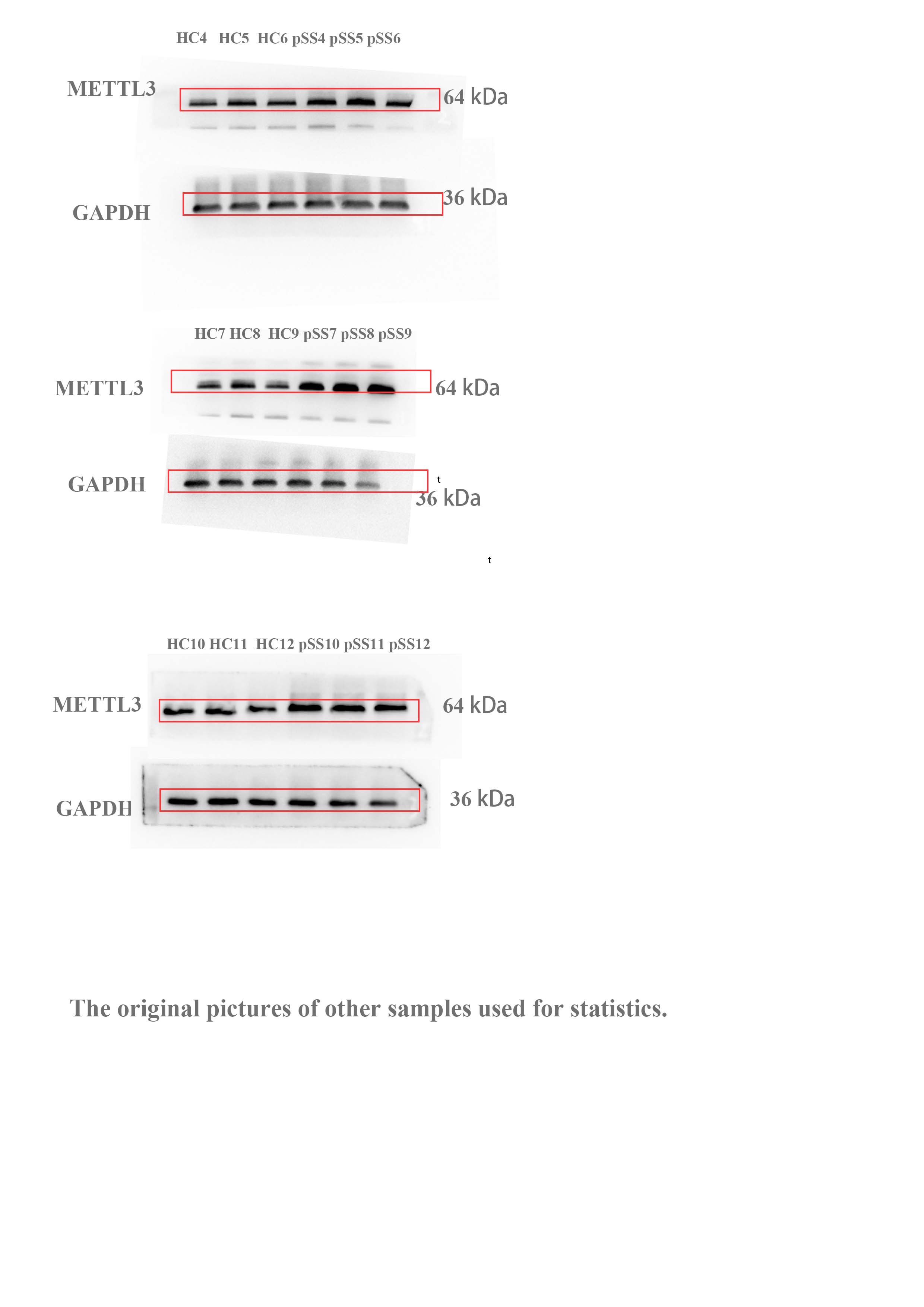


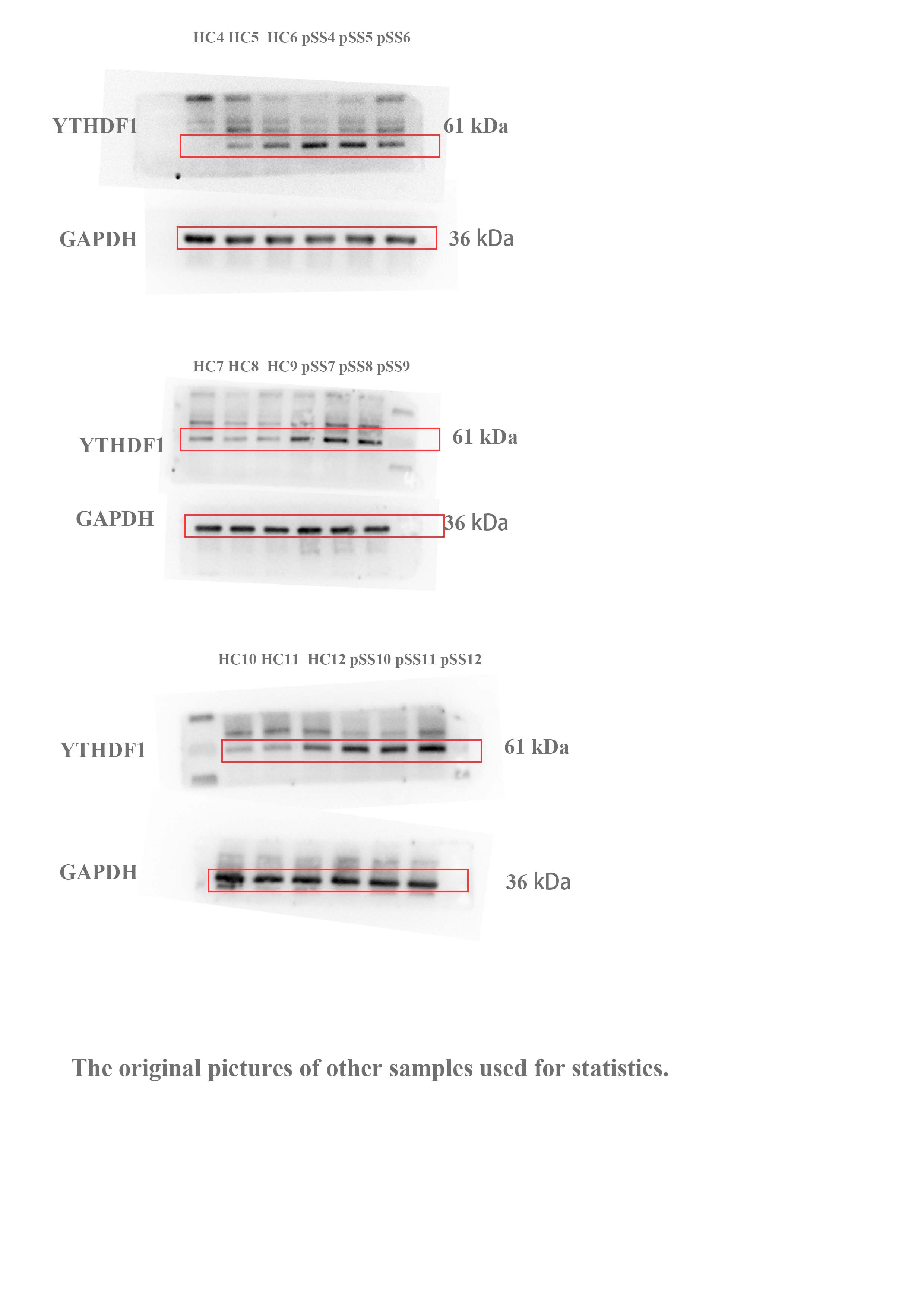

Supplement: Supplementary file 1 — Additional file 1: Fig. 1. The correlation analyses of m6A RNA level with the METTL3 mRNA level in the PBMCs of health controls. Fig. 2. Representative raw images showing METTL3 and YTHDF1 expression in the different group [file 12886_2023_2988_MOESM1_ESM.docx]
